# Field-induced reactant enrichment enhances benzyl alcohol electrooxidation coupled with hydrogen evolution†

**DOI:** 10.1039/d5sc03582a

**Published:** 2025-07-14

**Authors:** Yifan Yan, Lina Chen, Shaoyu Kang, Xiaofei Li, Claire Coulthard, Jianqin Tang, Chunping Chen, Zhenhua Li, Mingfei Shao, Dermot O'Hare

**Affiliations:** a State Key Laboratory of Chemical Resource Engineering, Beijing University of Chemical Technology Beijing 100029 P. R. China shaomf@mail.buct.edu.cn; b Department of Chemistry, University of Oxford, Chemistry Research Laboratory Oxford OX1 3TA UK dermot.ohare@chem.ox.ac.uk; c Quzhou Institute for Innovation in Resource Chemical Engineering Quzhou 324000 P. R. China

## Abstract

Electrochemical hydrogen evolution coupled with organic oxidation (EHCO) offers a promising route to improve the energy efficiency of water electrolysis by replacing the sluggish oxygen evolution reaction with value-added organic oxidation processes. However, the limited adsorption of organic reactants on the catalyst surface remains a key bottleneck, constraining the overall performance of EHCO systems. Herein, we report a field-induced enrichment strategy to enhance benzyl alcohol electrooxidation coupled with hydrogen evolution. A nanostructured cooperative catalyst composed of Au nanoparticles supported on copper oxide nanowires (Au/CuO NWs) delivers an impressive current density of 734 mA cm^−2^ at 1.5 V *vs.* RHE, along with a benzyl alcohol oxidation rate of 4.74 mmol cm^−2^ h^−1^ and a 91% faradaic efficiency for benzoic acid production. The catalyst demonstrated excellent stability and sustained industrial-level current output (>300 mA cm^−2^) in a membrane-free flow electrolyser. Combined experimental and COMSOL simulation results reveal that the nanowire morphology induces stronger localised electric fields, promoting the interfacial enrichment of benzyl alkoxide and the formation of OH* species, thereby improving the overall performance. This work establishes a new paradigm for leveraging local electric field effects in electrocatalyst design, advancing the development of next-generation EHCO systems.

## Abstract 2

Field-induced enrichment on Au/CuO nanowires boosts electrochemical hydrogen evolution coupled with benzyl alcohol oxidation, achieving efficient co-production of hydrogen and value-added chemicals.

## Graphical abstract

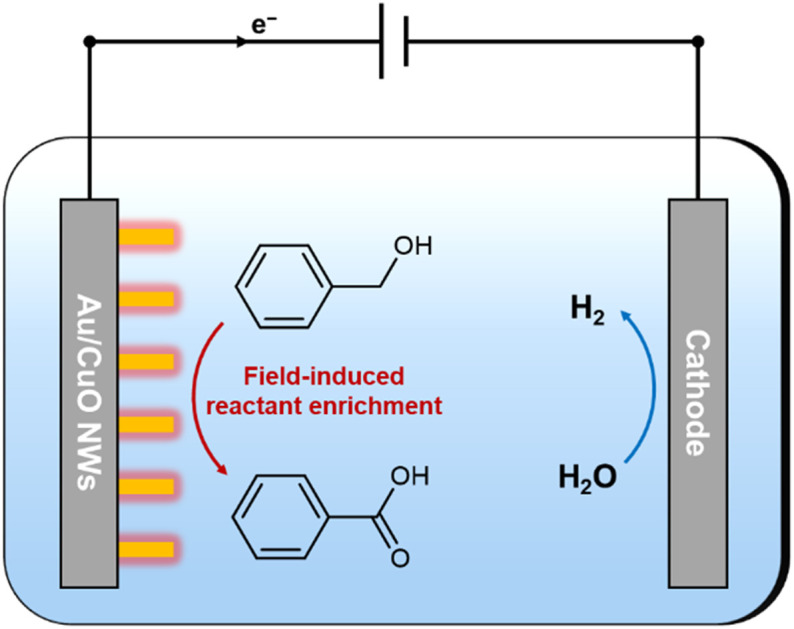

## 1 Introduction

The global transition toward sustainable energy has driven growing interest in green hydrogen technologies. Among various approaches, water electrolysis is widely recognised as a clean and efficient method for producing high-purity hydrogen, powered by renewable electricity.^1–3^ This carbon-neutral process offers a scalable solution for energy storage and conversion, addressing both global energy and environmental challenges. However, its widespread deployment is constrained by the inherently sluggish kinetics of the anodic oxygen evolution reaction (OER), a multi-step four-electron process that requires high overpotentials. In addition, the generation of low-value O_2_, along with associated safety concerns and system complexity, undermines its industrial appeal.^4–6^

Previous studies have demonstrated that replacing the sluggish OER with thermodynamically more favourable organic oxidation reactions can substantially reduce the anodic overpotential, while simultaneously yielding high-value-added chemicals.^7–9^ This dual advantage not only enhances the overall energy efficiency of electrolysis systems but also improves the economic feasibility of hydrogen production. Therefore, electrochemical hydrogen evolution coupled with organic oxidation (EHCO) has garnered significant attention as a promising strategy to overcome the limitations of conventional water electrolysis in terms of both energy consumption and cost-effectiveness.^10^

Among various organic substrates, benzyl alcohol (PhCH_2_OH) is an attractive feedstock owing to its efficient electrooxidation to benzoic acid (PhCOOH), a high-value chemical widely used in the production of polymers, preservatives, and pharmaceuticals.^11^ The electrooxidation of benzyl alcohol typically proceeds through a multistep reaction pathway, involving substrate adsorption on the catalyst surface, the generation of reactive oxygen species (*e.g.*, OH*), catalytic transformation, and subsequent product desorption.^12–14^ Despite increasing interest, most studies have focused on improving the intrinsic activity of catalytic sites, with relatively less attention paid to reactant adsorption.^15–17^ This oversight shifts the rate-determining step to reactant adsorption, thereby limiting the overall catalytic performance. Duan and co-workers recently addressed this challenge by constructing a cooperative catalyst (Au/CoOOH), wherein the Au sites promote benzyl alcohol adsorption through σ-π interaction, and the CoOOH component facilitates OH* generation.^12^ This bifunctional synergy enabled a remarkable current density of 540 mA cm^−2^ at 1.5 V *vs.* RHE. More recently, Chen and co-workers reported that PtZn-ZnO_*x*_ interface engineering not only enhanced the adsorption of PhCH_2_OH, but also accelerated OH* generation due to the unsaturated coordination environment of Zn atoms, thereby achieving outstanding performance.^18^ Despite these advances, further enhancement in the adsorption and enrichment of benzyl alcohol at the catalyst surface remains a critical challenge.

Field-induced reagent enrichment, a concept originally demonstrated in CO_2_ electroreduction, offers a potential solution to this limitation. High-curvature nanostructures can generate enhanced local electric fields that concentrate reactive species such as K^+^ and CO_2_ at the catalyst surface, thereby improving conversion rates and selectivity.^19,20^ For example, gold nanoneedles enabled a CO_2_ reduction current density of 22 mA cm^−2^ with over 95% faradaic efficiency for CO production, outperforming conventional Au nanoparticles by an order of magnitude.^19^ This concept has recently been extended to other electrochemical systems.^21^ In particular, localised enhanced electric fields have been shown to facilitate OH^−^ adsorption and thereby promote OER performance.^22^ Inspired by these findings, we hypothesise that field-induced enrichment could similarly enhance the adsorption of benzyl alcohol. Notably, under strongly alkaline conditions, benzyl alcohol predominantly exists in the form of benzyl alkoxide anions (PhCH_2_O^−^), which are more susceptible to electric field modulation.^23^ Inspired by these insights, we propose a field-induced enrichment strategy to substantially improve the performance of benzyl alcohol electrooxidation coupled with hydrogen evolution.

Herein, we successfully apply the concept of field-induced reactant enrichment to enhance electrochemical hydrogen evolution coupled with benzyl alcohol oxidation. Specifically, we design a nanostructured cooperative catalyst comprising Au nanoparticles supported on copper oxide nanowire arrays (Au/CuO NWs). An impressive current density of 734 mA cm^−2^ was achieved at 1.5 V *vs.* RHE, surpassing most previously reported systems for benzyl alcohol electrooxidation. The corresponding oxidation rate reaches 4.74 mmol cm^−2^ h^−1^, which is 3.6 and 5.1 times higher than that of Au supported on CuO nanosheets (Au/CuO NSs) and nanoparticles (Au/CuO NPs), respectively, and 9.5 times higher than that of pure Au. The experimental results and multiphysics simulations (COMSOL) reveal that the enhanced local electric fields generated by the nanowire architecture facilitate the interfacial enrichment of benzyl alcohol and promote the formation of OH*, thereby accelerating the overall oxidation kinetics. This work offers a new paradigm for integrating intrinsic physical field effects with organic electrooxidation, paving the way toward the development of high-performance EHCO systems.

## 2 Results and discussion

### 2.1 Characterisation of multiple morphology Au/CuO cooperative catalysts

Our recent work has revealed that the Au/CuO cooperative catalyst contains abundant oxygen vacancies, which serve as adsorption sites to enhance the adsorption of benzyl alcohol.^24^ As a result, the Au/CuO catalyst exhibits superior catalytic performance compared to Au/CoOOH. Motivated by these findings, we selected Au/CuO as the model cooperative catalyst to investigate the effect of field-induced reactant enrichment. Due to the variation in local electric field strength induced by different morphologies,^19^ three different CuO nanostructures; nanoparticles (NPs), nanosheets (NSs), and nanowires (NWs), were synthesised on Cu foam (detailed synthesis methods are provided in the SI). These were used to prepare three distinct morphology Au/CuO cooperative catalysts (Au/CuO NPs, Au/CuO NSs, and Au/CuO NWs) by electrodepositing Au nanoparticles onto the corresponding CuO substrates. This experimental design enables direct elucidation of the contribution of nanostructure-induced local electric fields to the adsorption and activation of benzyl alcohol during electrooxidation. For comparison, pure Au nanoparticles and pure CuO were also synthesised on Cu foam following the same methods.

**Fig. 1:**
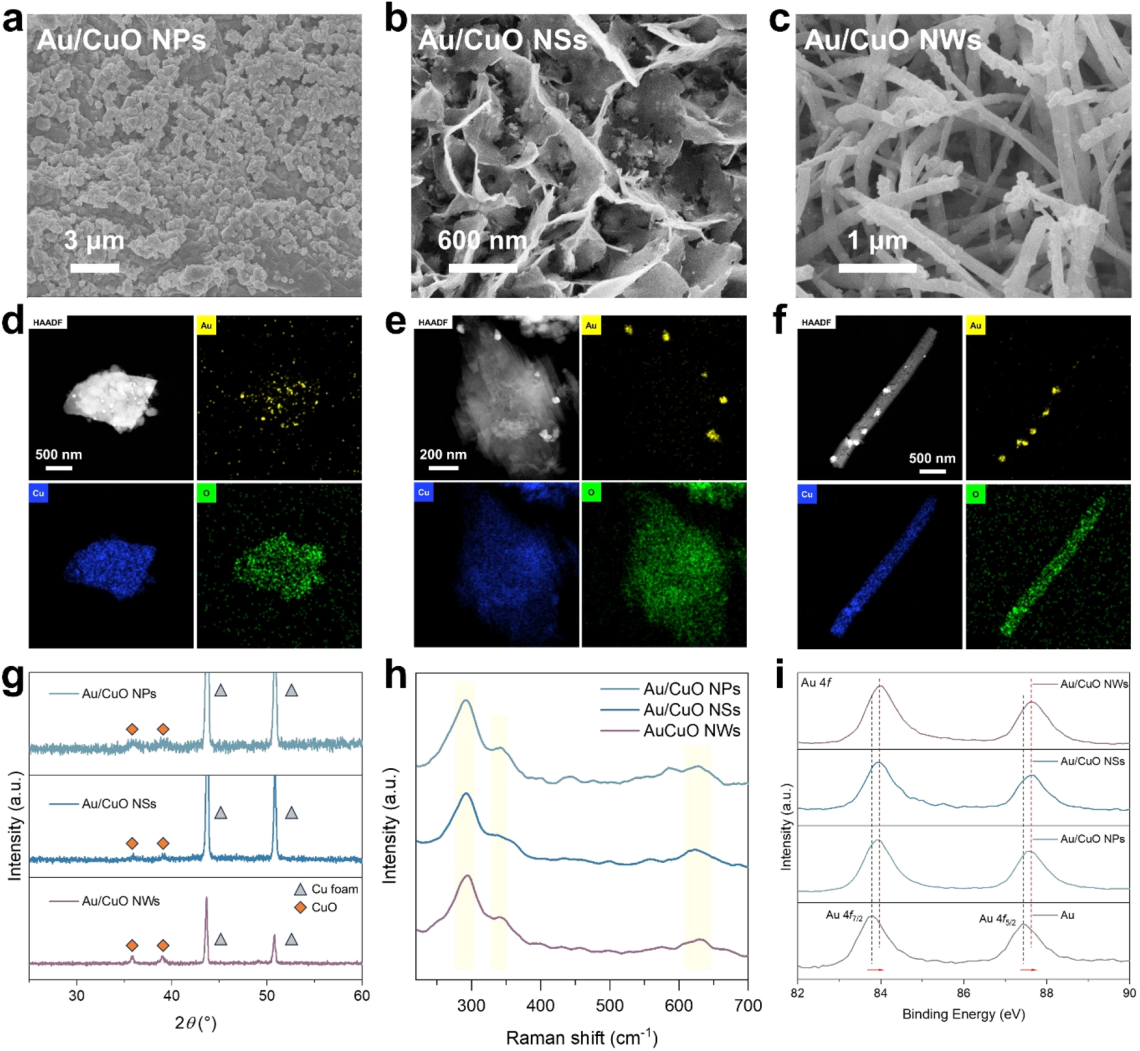
SEM images of (a) Au/CuO NPs, (b) Au/CuO NSs and (c) Au/CuO NWs. EDS mapping of (d) Au/CuO NPs, (e) Au/CuO NSs and (f) Au/CuO NWs. (g) XRD patterns of three Au/CuO cooperative catalysts. (h) Raman spectra of three Au/CuO cooperative catalysts. (i) High-resolution Au 4f XPS spectra of three Au/CuO cooperative catalysts and pure Au.

The three synthesised Au/CuO cooperative catalysts were systematically characterised to investigate their morphological features. Scanning electron microscopy (SEM) images reveal distinct surface architectures for each sample. The Au/CuO NPs display a rough, granular morphology composed of densely packed nanoparticles (Fig. 1a). In contrast, the Au/CuO NSs exhibit vertically aligned and interconnected nanosheets with abundant open spaces (Fig. 1b). Notably, small protruding particles can be observed on the surface of the nanosheets, which are attributed to the Au nanoparticles. The Au/CuO NWs consist of vertically oriented nanowires forming a dense and uniform array on the Cu foam (Fig. 1c and S1†). Similarly, small surface protrusions are also presented along the nanowires. Energy-dispersive spectroscopy (EDS) elemental mapping confirms that the Au nanoparticles are uniformly distributed across the surfaces of all three CuO morphologies (Fig. 1d–f). High-resolution transmission electron microscopy (HRTEM) reveals that the Au nanoparticles on all three CuO morphologies exhibit similar particle sizes (Fig. S2†), with average diameters of ∼40 nm for both Au/CuO NPs and Au/CuO NSs, and ∼50 nm for Au/CuO NWs. Moreover, inductively coupled plasma-atomic emission spectrometry (ICP-AES) measurements confirm that the Au loadings are comparable across the samples (Table S1†), further suggesting that the differences in catalytic performance are not due to variations in Au content or distribution. These results collectively demonstrate the successful fabrication of three well-defined morphology Au/CuO cooperative catalysts containing well-dispersed Au nanoparticles.

The structures of the catalysts were examined by X-ray diffraction (XRD, Fig. 1g). All samples exhibit two prominent Bragg peaks at 2*θ* = 43.7° and 50.8°, corresponding to the (111) and (200) planes of metallic Cu, respectively. Additionally, two low-intensity Bragg peaks at 2*θ* = 35.9° and 39.1° can be observed, which are assigned to the (−111) and (111) planes of monoclinic CuO.^25^ No distinct Bragg peaks corresponding to metallic Au were detected, likely due to the relatively low Au loading. Raman spectra were performed to further confirm the CuO phase (Fig. 1h). All three Au/CuO samples exhibit characteristic CuO vibrational bands at 291, 341, and 630 cm^−1^, which correspond to the A_g_ (296 cm^−1^), B_g_(1) (346 cm^−1^), and B_g_(2) (636 cm^−1^) modes of monoclinic CuO, respectively.^26^

The electronic interactions between Au and CuO were investigated by X-ray photoelectron spectroscopy (XPS). As shown in Fig. 1i, all three Au/CuO cooperative catalysts exhibit positive shifts in the binding energies of the Au 4f peaks compared to pure Au, while the Cu 2p peaks show a shift toward lower binding energies relative to pristine CuO (Fig. S3†). These shifts indicate electronic interactions at the Au–CuO interface, with electrons transferring from Au to CuO. Notably, no significant differences are observed in the Au 4f or Cu 2p binding energies among the three Au/CuO samples. Collectively, these results suggest that aside from their morphological differences, the three Au/CuO cooperative catalysts exhibit comparable crystalline phase and electronic structure.

### 2.2 Benzyl alcohol electrooxidation performance

Electrochemical measurements were carried out in a standard three-electrode configuration using a glass cell, with a Pt foil as the counter electrode and Ag/AgCl electrode as the reference electrode. The electrooxidation performance of benzyl alcohol on the prepared catalysts was initially evaluated using linear sweep voltammetry (LSV). In 1 M KOH (Fig. S4†), all samples exhibited an oxidation peak between 0.8 and 1.0 V *vs.* RHE, which is attributed to the redox transition of Cu species in the Cu foam (Cu^0^/Cu^+^ to Cu^2+^),^27^ as well as the onset of the oxygen evolution reaction (OER).

**Fig. 2:**
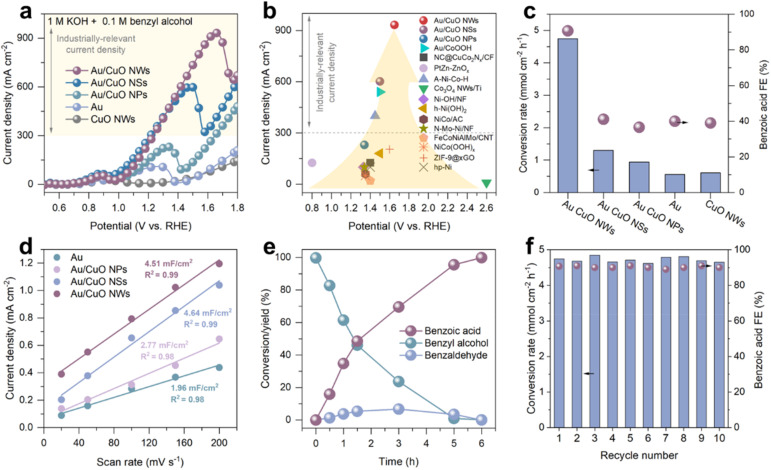
(a) LSV curves of benzyl alcohol electrooxidation at scan rate of 10 mV s^−1^ in 1 M KOH with 0.1 M benzyl alcohol over different catalysts. (b) Current densities of benzyl alcohol electrooxidation in the literature and in this work. (c) Conversion rate of benzyl alcohol and the corresponding FE of benzoic acid over different catalysts. (d) The charging currents measured plotted as a function of scan rate. (e) Kinetic curves for benzyl alcohol oxidation over Au/CuO NWs at 1.5 V *vs.* RHE. (f) Stability test of Au/CuO NWs for benzyl alcohol oxidation in batch reaction (10 batches, 10 h).

Upon the addition of 0.1 M benzyl alcohol (Fig. 2a), bare CuO NWs exhibited negligible oxidation activity below 1.3 V *vs.* RHE (Fig. S5†). A sharp increase in current density was observed beyond 1.35 V *vs.* RHE, corresponding to the oxidation of benzyl alcohol by CuOOH species generated *in situ*.^28,29^ In contrast, both Au and all Au/CuO catalysts exhibited a pronounced oxidation current starting at ∼0.9 V *vs.* RHE, which is consistent with the potential required for OH* formation on Au (0.85 V *vs.* RHE) and significantly lower than the CuOOH formation potential. This indicates that Au acts as the primary catalytic site for OH* generation in the Au/CuO system, facilitating the electrooxidation of benzyl alcohol, in consist with our recent findings. Notably, all Au/CuO samples delivered significantly higher current densities than pure Au or CuO, underscoring the advantage of the cooperative catalyst design. Among the three Au/CuO catalysts, the current density decreases in the order of Au/CuO NWs, Au/CuO NSs, and Au/CuO NPs, highlighting the critical role of nanostructure morphology on catalytic performance. Remarkably, the Au/CuO NWs achieved a maximum current density of 933 mA cm^−2^, outperforming most previously reported electrocatalysts for benzyl alcohol oxidation (Fig. 2b and Table S1†). We also compared the benzyl alcohol oxidation performances of Au/CuO cooperative catalysts with commercial Pt/C and RuO_2_ catalysts. The results show that the current density of the Au/CuO cooperative catalysts is significantly higher than that of commercial catalysts (Fig. S6†).

To further evaluate the catalytic efficiency and product selectivity, chronoamperometric (CA) measurements were conducted, and the reaction products were quantitatively analysed by high-performance liquid chromatography (HPLC). At 1.5 V *vs.* RHE (Fig. S6†), Au/CuO NWs achieved a benzyl alcohol conversion rate of 4.74 mmol cm^−2^ h^−1^, which is 3.6 and 5.1 times higher than that of Au/CuO NSs and Au/CuO NPs, respectively, and 8.5 times greater than that of pure Au (Fig. 2c). In addition, a faradaic efficiency (FE) of 91% toward benzoic acid was achieved with Au/CuO NWs (Fig. S7†), significantly outperforming other samples. We further evaluated the electrochemically active surface area (ECSA) of pure Au and the three Au/CuO catalysts by cyclic voltammetry (CV) at different scan rates (Fig. S8†).^30^ As shown in Fig. 2d and S9,† both Au/CuO NWs and Au/CuO NSs exhibit significantly higher ECSA compared to Au/CuO NPs and pure Au. However, the ECSA of Au/CuO NWs and Au/CuO NSs are comparable, despite the substantially higher catalytic activity observed for Au/CuO NWs. These results suggest that the superior performance of Au/CuO NWs cannot be solely attributed to the increased ECSA. Instead, the enhanced activity is likely driven by the field-induced reactant enrichment effect, which was considered in the catalyst design.

To evaluate the greater practical catalytic performance of Au/CuO NWs, the reaction was extended until the conversion of benzyl alcohol exceeded 99%. The kinetic curves show that the yield of benzoic acid reaches 99% (Fig. 2e), indicating that the produced benzoic acid remains stable without undergoing further oxidation. Notably, benzaldehyde was initially observed as a transient by-product. However, as the reaction proceeded, it was progressively oxidised to benzoic acid, demonstrating that benzoic acid is the final and dominant product.

We subsequently evaluated the stability of the Au/CuO NWs catalyst over ten consecutive reaction batches, corresponding to a total reaction time of 10 hours. As shown in Fig. 2f, both the benzyl alcohol conversion and the high FE toward benzoic acid were largely maintained, together with the preservation of the original nanowire structure (Fig. S10†), demonstrating the excellent stability of the catalyst under continuous operation. Moreover, we employed a pulsed potential strategy to achieve stable, high-current-density (>300 mA cm^−2^) operation for 12 hours (Fig. S12b†).^31^

Previous studies have shown that Au-based catalysts tend to deactivate during long-term reaction due to surface oxidation into inactive AuO_*x*_ species, which can be mitigated by enhancing local reactant concentrations.^7,32^ Therefore, we further investigated the long-term stability of Au/CuO NPs and Au/CuO NWs. As shown in Fig. S11,† Au/CuO NPs exhibited rapid deactivation at 1.35 V *vs.* RHE, with the current density sharply dropping from 250 mA cm^−2^ to negligible 2 mA cm^−2^ within 6000 s. In contrast, Au/CuO NWs demonstrated significantly slower degradation, retaining a current density of 150 mA cm^−2^ even after 36 000 s of continuous reaction. This remarkable enhancement in stability is likely attributed to the nanowire architecture of Au/CuO NWs, which induces a stronger local electric field. The intensified field facilitates the adsorption and enrichment of benzyl alcohol, thereby effectively mitigating deactivation over extended periods.

### 2.3 Mechanistic studies of the enhanced activity of Au/CuO NWs

**Fig. 3:**
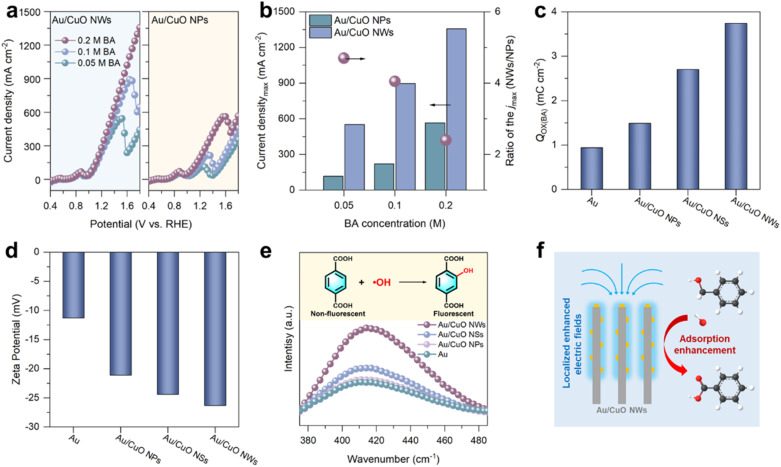
(a) LSV curves of Au/CuO NPs and Au/CuO NWs at scan rate of 10 mV s^−1^ in 1 M KOH solution with different concentrations of benzyl alcohol (BA). (b) Maximum current density of Au/CuO NWs and Au/CuO NPs at different benzyl alcohol concentrations and the corresponding ratio of *j*_max_. (c) Values of the oxidation charges (*Q*_Ox_(BA)) for the adsorbed benzyl alcohol over Au/CuO samples and pure Au. (d) Zeta potential of Au/CuO samples and pure Au. (e) Fluorescence spectra for the detected ˙OH radicals in electrolyte using terephthalic acid (TPA, 0.4 mM) as a probe molecule. (f) Schematic illustration of nanowire-induced localised electric field facilitating interfacial adsorption enrichment of benzyl alcohol.

We have focused on elucidating the underlying mechanism behind the exceptional performance of Au/CuO NWs. To this end, LSV were performed at varying benzyl alcohol concentrations, selecting Au/CuO NWs and Au/CuO NPs as representative samples for comparison. As shown in Fig. 3b, both the current density and deactivation potential of the two catalysts increased with rising benzyl alcohol concentration. However, Au/CuO NWs consistently exhibited a significantly higher current density and a more positive deactivation potential than Au/CuO NPs under identical conditions. Notably, the ratio of the maximum current densities between Au/CuO NWs and Au/CuO NPs decreased as the benzyl alcohol concentration increased. These results suggest that Au/CuO NWs exhibit a stronger capacity for adsorbing and enriching benzyl alcohol, thereby enhancing catalytic performance and improving resistance to deactivation.

**Fig. 4:**
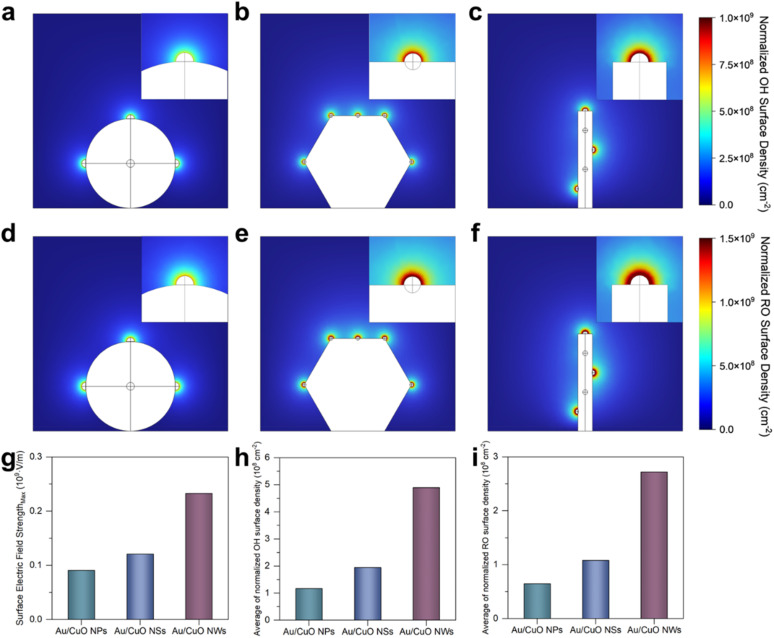
Simulated normalised OH^−^ surface density distribution on (a) Au/CuO NPs, (b) Au/CuO NSs and (c) Au/CuO NWs. Simulated distribution of normalised benzyl alkoxide (RO) surface density on (d) Au/CuO NPs, (e) Au/CuO NSs and (f) Au/CuO NWs. (g) The maximum surface electric field strength of three Au/CuO catalysts. (h) The average of normalised OH^−^ surface density on three Au/CuO catalysts. (i) The average of normalised RO surface density on three Au/CuO catalysts.

To further assess the catalyst's ability to adsorb benzyl alcohol, open circuit potential (OCP) measurements were conducted to probe changes in the organic adsorbate content within the inner Helmholtz layer.^33^ Upon injecting 0.05 M benzyl alcohol, Au/CuO NWs exhibited a significantly larger OCP shift compared to other Au/CuO samples and pure Au, indicating a higher benzyl alcohol adsorption capacity within the inner Helmholtz layer (Fig. S12†). This enhanced adsorption behaviour was further validated by electrochemical adsorbate-stripping (EAS) experiment.^34^ The oxidation charges of benzyl alcohol (*Q*_Ox(BA)_) can be used to quantify the amount of adsorbed reactant (Fig. S13†). As shown in Fig. 4b, the *Q*_Ox(BA)_ value of Au/CuO NWs reached 3.7 mC cm^−2^, which is markedly higher than those of Au/CuO NSs (2.7 mC cm^−2^), Au/CuO NPs (1.5 mC cm^−2^), and pure Au (0.9 mC cm^−2^), confirming the superior affinity of Au/CuO NWs for benzyl alcohol. Importantly, both the OCP and EAS measurements reveal a trend in which Au/CuO NWs exhibit stronger adsorption than Au/CuO NSs, followed by Au/CuO NPs. This sequence closely matches the catalytic activity observed across the samples (Fig. 2a). Such a direct correlation demonstrates that the nanostructure morphology can modulate the adsorption capability of the catalyst toward benzyl alcohol, thereby playing a crucial role in enhancing electrocatalytic performance.

Earlier research has elucidated that Au-based catalysts facilitate alcohol electrooxidation by adsorbing OH^−^ to form active oxygen species (OH*), which are essential for the oxidation process.^8^ Notably, localised electric field enhancement has been reported to promote OH^−^ adsorption at the catalyst surface. To investigate this effect, we compared the OH^−^ adsorption capabilities of Au and various Au/CuO catalysts using zeta potential measurements.^35^ As shown in Fig. 3d, Au/CuO NWs exhibit the most negative zeta potential among all samples, indicating a stronger ability to accumulate OH^−^ within the inner Helmholtz layer. Since the adsorbed OH^−^ can be electrooxidised to generate OH*, we further conducted fluorescence probe experiments to quantitatively evaluate OH* generation (Fig. S14a†).^36^ As shown in Fig. 3e, the fluorescence intensity observed for Au/CuO NWs is significantly higher than that of other Au/CuO samples and pure Au, suggesting that Au/CuO NWs is more conducive to generating OH*. After adding benzyl alcohol, a notable decrease in fluorescence intensity was observed for Au/CuO NWs (Fig. S14b†), confirming that the generated OH* species actively participate in benzyl alcohol electrooxidation.

Importantly, both the zeta potential and fluorescence probe results reveal a consistent trend across the three Au/CuO catalysts, with Au/CuO NWs exhibiting the highest value, followed by Au/CuO NSs and Au/CuO NPs. This trend aligns well with their benzyl alcohol adsorption capacities. These findings indicate that nanostructure-induced variations in local electric field strength can modulate the adsorption of both OH^−^ and benzyl alcohol, ultimately influencing the overall electrocatalytic performance. Taken together, the superior catalytic performance of Au/CuO NWs can be attributed to the nanowire architecture, which induces a stronger localised electric field. This enhanced field significantly promotes the adsorption and activation of OH^−^ (*i.e.*, OH* formation) and facilitates the efficient interfacial enrichment of benzyl alcohol, thereby contributing to the outstanding electrocatalytic performance (Fig. 3f).

### 2.4 COMSOL simulations

To further validate the experimental observations, COMSOL simulations were employed to model the effects of local electric field enhancement and interfacial species enrichment (see Experimental section for details).^21,22^ The models were constructed based on the SEM and TEM results (Fig. 1), with five Au nanoparticles uniformly distributed on each type of CuO substrate. Owing to the three-dimensional nature of the models, only three Au nanoparticles are visible in the two-dimensional projections of the Au/CuO NPs and Au/CuO NWs models (Fig. S15†). However, this does not compromise the reliability of the simulation results. As shown in Fig. 4g, the maximum surface electric field strength of Au/CuO NWs is higher than that of Au/CuO NSs, which in turn exceeds that of Au/CuO NPs. This trend is consistent with the original design rationale of the catalysts.

We further analysed the surface density of reactive species, including OH^−^ (Fig. 4a–c) and benzyl alcohol alkoxide (Fig. 4d–f), across the three Au/CuO models. As shown in Fig. 4h and i, the average surface densities of both OH^−^ and benzyl alkoxide exhibit the same trend, with Au/CuO NWs showing the highest values, followed by Au/CuO NSs and Au/CuO NPs. This trend is in excellent agreement with the experimental results (Fig. 3). Notably, the enhanced accumulation of OH^−^ at the catalyst surface can directly accelerate the generation of OH*, a key reactive intermediate in the electrooxidation process. The strong localised electric field, induced by the nanowire architecture, lowers the energy barrier for OH^−^ activation, thereby promoting OH* formation.

These simulation results provide visual support for the mechanistic hypothesis proposed in Fig. 3f. Specifically, the superior catalytic performance of Au/CuO NWs can be attributed to nanowire architecture, which induces a stronger localised electric field. This enhanced field facilitates the adsorption and activation of OH^−^ and promotes the interfacial enrichment of benzyl alcohol, thereby improving the overall performance.

To evaluate the generality of the strategy, we further tested ethanol and cyclohexanol as substrates. The results show that Au/CuO NWs exhibit better performance than Au/CuO NPs (Fig. S18†). However, the observed current densities are substantially lower than those for benzyl alcohol oxidation. This is likely due to the higher p*K*_a_ values of ethanol and cyclohexanol, which limit the formation of alkoxide species under alkaline conditions and consequently reduce the effectiveness of electric field-induced interfacial enrichment. In future studies, we plan to employ Kelvin probe force microscopy (KPFM) or electrostatic force microscopy to directly visualise the localised electric field distribution at the catalyst surface. In addition, *in situ* spectroscopic techniques will be applied to capture the dynamic evolution of surface species, enabling deeper mechanistic understanding of the field-induced enrichment process.

### 2.5 Flow electrolyser studies

**Fig. 5:**
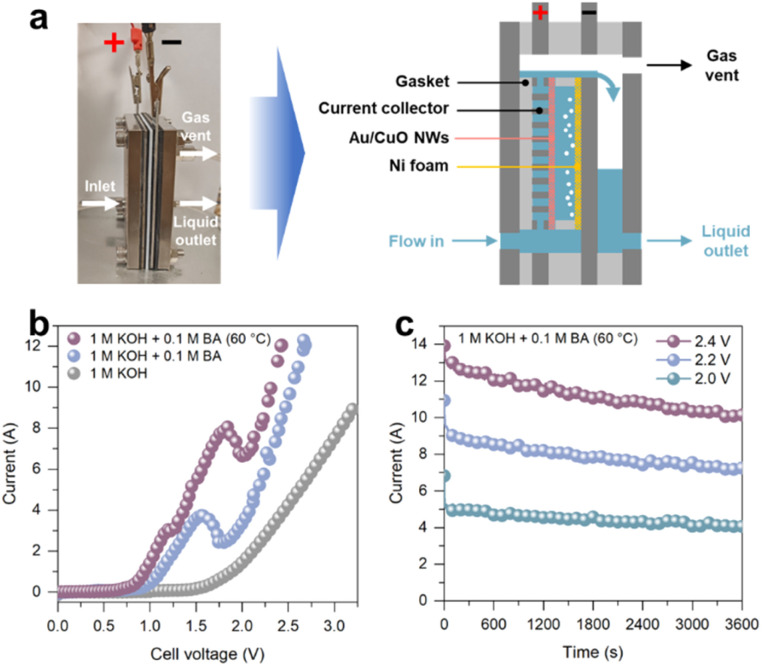
(a) Photograph and schematic illustration of the membrane-free flow electrolyser. (b) The LSV curves of Au/CuO NWs in 1 M KOH with or without 0.1 M benzyl alcohol (BA) in the membrane-free flow electrolyser. (c) *I*–*t* curves of Au/CuO NWs catalyst at 2.0 V, 2.2 V and 2.4 V in 1 M KOH containing 0.1 M benzyl alcohol at 60 °C in the membrane-free flow electrolyser.

To evaluate the catalyst under more practical conditions, we carried out two-electrode tests in a homemade membrane-free flow electrolyser using Au/CuO NWs as the anode and Ni foam as the cathode, with a working area of 30 cm^2^ (Fig. 5a and S16†). This configuration enables continuous liquid flow and gas release, providing a more industrially relevant testing environment. As shown in the LSV curves (Fig. 5b), the onset potential for water splitting in 1 M KOH was ∼1.55 V. Upon the introduction of 0.1 M benzyl alcohol, the onset potential significantly decreased to ∼0.85 V, confirming the thermodynamic advantage of the benzyl alcohol oxidation reaction over the oxygen evolution reaction. Moreover, increasing the reaction temperature to 60 °C further reduced the onset potential to 0.75 V and enhanced the absolute current, indicating that elevated temperature accelerates benzyl alcohol oxidation kinetics. CA measurements were subsequently conducted at different cell voltages to evaluate the catalytic performance. As shown in Fig. 5c, the absolute current for benzyl alcohol oxidation increased with cell voltage from 2.0 V to 2.4 V. Notably, at 2.4 V, we achieved electrochemical hydrogen evolution coupled with benzyl alcohol oxidation at industrially relevant current densities (>300 mA cm^−2^, total current > 9 A), demonstrating the practical applicability of this strategy.

The successful demonstration of Au/CuO NWs in a membrane-free flow electrolyser highlights the potential for practical and scalable application. Although noble metal Au is used, the catalyst remains cost-effective due to its low loading and uniform dispersion on inexpensive CuO supports. The membrane-free configuration also simplifies system design and reduces overall cost, enhancing industrial applicability. These advantages, together with the high current density and operational stability, support the feasibility of applying this EHCO strategy in decentralised hydrogen production and organic oxidation. Future efforts will aim to reduce noble metal usage through controlled deposition, alloying, or single-atom approaches. Additionally, a techno-economic analysis (TEA) will be carried out to quantitatively evaluate the system's cost-performance balance and scalability, guiding further development toward real-world implementation in sustainable energy and chemical manufacturing.

## 3 Conclusions

In summary, we have developed a field-induced enrichment strategy to significantly enhance the performance of electrochemical hydrogen evolution coupled with benzyl alcohol oxidation. By constructing a nanostructured Au/CuO cooperative catalyst with nanowire morphology, we achieved efficient interfacial enrichment of both OH^−^ and benzyl alkoxide species, thereby accelerating the overall oxidation kinetics. A maximum current density of 933 mA cm^−2^ was achieved with the Au/CuO NWs catalyst, outperforming the majority of reported systems for benzyl alcohol oxidation. Experimental measurements combining COMSOL simulations confirmed that the enhanced performance originated from the nanowire-induced localised electric fields, which promoted reactant adsorption and OH* generation. Furthermore, the catalyst demonstrated excellent stability and sustained industrial-level current output (>300 mA cm^−2^) in a membrane-free flow electrolyser. This work not only provides a viable approach for overcoming kinetic limitations in EHCO systems but also offers a general strategy for integrating physical field effects into catalyst design for green energy conversion.

## Author contributions

Yifan Yan: conceptualisation, methodology, writing original draft, writing – review & editing, visualisation, validation, formal analysis, investigation, and data curation. Lina Chen: validation, formal analysis, investigation, and data curation. Shaoyu Kang: visualisation, validation, formal analysis, investigation, and data curation. Xiaofei Li: formal analysis and data curation. Claire Coulthard: formal analysis and data curation. Jianqin Tang: formal analysis and data curation. Chunping Chen: formal analysis, resources, data curation and investigation. Zhenhua Li: conceptualisation, methodology, supervision. Mingfei Shao: conceptualisation, supervision and project administration. Dermot O'Hare: conceptualisation, methodology, writing – review & editing, supervision and project administration.

## Conflicts of interest

There are no conflicts to declare.

## Supplementary Material

SC-OLF-D5SC03582A-s001

## Data Availability

The data that support the plots within the manuscript and other findings of this study are available from the corresponding author upon reasonable request.
