# Supplementary material for: Field-induced reactant enrichment enhances benzyl alcohol electrooxidation coupled with hydrogen evolution
Source: Chem Sci. 2025 Jul 14;16(32):14646–54. doi: 10.1039/d5sc03582a (PMC12257163; doi:10.1039/d5sc03582a)
Supplement: SC-016-D5SC03582A-s001 [file SC-016-D5SC03582A-s001.pdf]

Supplementary Information (SI) for Chemical Science.
This journal is © The Royal Society of Chemistry 2025

Supplementary Information

Field-Induced Reactant Enrichment Enhances Benzyl Alcohol Electrooxidation Coupled with Hydrogen Evolution

Yifan Yan,‡[a,b] Lina Chen,‡[b] Shaoyu Kang,‡[b] Xiaofei Li,[b] Claire Coulthard,[b] Jianqin Tang,[b] Chunping Chen,[b] Zhenhua Li,[a,c] Mingfei Shao,*[a,c] Dermot O'Hare*[b]

[a]State Key Laboratory of Chemical Resource Engineering, College of Chemistry, Beijing University of Chemical Technology, Beijing 100029, China

[b] Department of Chemistry, University of Oxford, Chemistry Research Laboratory, Oxford OX1 3TA, U.K.

[c]Institute for Innovation in Resource Chemical Engineering, Quzhou 324000, China

‡Yifan Yan, Lina Chen and Shaoyu Kang contributed equally to this work

*Correspondence and requests for materials should be addressed to M.S. (email: *shaomf@mail.buct.edu.cn*) and D.O.H (email: *dermot.ohare@chem.ox.ac.uk*).

Methods

Synthesis of CuO nanoparticles (NPs).

Copper foam (CF) was used as matrix for growing CuO nanosheets. Initially, a piece of CF (25 × 40 × 1.5 mm) was sequentially washed with dilute HCl (2 M), ethanol, and deionized water (each for 15 min) to remove surficial oxides and contaminants. To prepare the electrodeposition solution, 0.2 M tartaric acid was first dissolved in deionized water, followed by the addition of 0.2 M $CuSO_4·5H_2O$. The solution was continuously stirred in an ice-water bath until it cooled to room temperature, during which 2.5 M NaOH was slowly added. CuO NPs was electrodeposited in a two-electrode system, with a Pt foil as the counter electrode. Cathodic deposition was carried out under a constant current density of 8 mA cm^{-2} for 30 min.

Synthesis of CuO nanosheets (NSs).

A piece of CF (35 × 30 × 1.5 mm) was initially pretreated as described above. Subsequently, 16 mL of NaOH solution (12 M) was mixed with 16 mL of ammonium persulfate (APS) solution (2.4 M). The mixture, along with the CF, was placed into a 25 mL beaker and sealed with a membrane. The system was then allowed to stand at room temperature for 12 hours to obtain CuO NSs. The resulting sample was thoroughly rinsed with anhydrous ethanol and deionized water five times, followed by vacuum drying at 70 °C for 12 hours.

Synthesis of CuO nanowires (NWs).

A piece of CF (35 × 30 × 1.5 mm) was initially pretreated as described above. Then, 0.913 g of ammonium persulfate (APS) was dissolved in 22 mL of deionized water under stirring. Separately, 10 M NaOH solution was prepared by dissolving NaOH in 8 mL of deionized water. The NaOH solution was then slowly added to the APS solution under continuous stirring to obtain a mixed solution. The pretreated copper foam was immersed in the resulting solution for 15 minutes to form $Cu(OH)_2$ NWs. The obtained $Cu(OH)_2$ NWs was dried in an oven at 60 °C for 2 hours, and then calcined in a muffle furnace by ramping the temperature to 180 °C at a rate of 5 °C/min, followed by heating at 180 °C for 2 hours to yield CuO NWs.

Synthesis of Au/CuO cooperative catalysts.

Au nanoparticles were electrodeposited on three CuO samples in a three-electrode configuration using saturated calomel electrode (SCE) reference electrode, Pt foil as counter electrode, and the prepared CuO samples as working electrode, respectively. The electrolyte was 0.1 M NaCl aqueous solution containing 5 mM $HAuCl_4$. Specifically, Au nanoparticles were deposited on CuO samples by stepping the potential to −0.6 V vs. SCE for 5 s, followed by stepping back to −0.2 V vs. SCE for 5 s for five cycles. The pure Au catalyst was prepared via the similar electrodeposition method by directly using Ni foam as the working electrode.

Characterizations.

X-ray diffraction patterns were collected on a Shimadzu XRD-6000 diffractometer using a Cu Kα source, with a scan range of 10−55° and scan step of 5° min^{-1}. X-ray photoelectron spectra (XPS) were performed on a Thermo VG ESCALAB 250 X-ray photoelectron spectrometer at a pressure of about 2×10^{-9} Pa using Al Kα X-rays as the excitation source. Scanning electrode microscope (SEM) images were recorded by a Zeiss SUPRA 55 Field Emission SEM with an accelerating voltage of 20 kV. Transmission electron microscopy (TEM) images were recorded with JEOL JEM-2010 high resolution (HR-)TEM with an accelerating voltage of 200 kV.

Electrochemical measurement.

All electrochemical measurements for benzyl alcohol oxidation were performed in 1 M KOH electrolyte at room temperature on an electrochemical workstation (CHI 760E, CH Instruments, Inc.). The powder catalysts (20% Pt/C and RuO_2) were dispersed in aqueous isopropanol solution with Nafion by sonification and sprayed on carbon fiber paper (CFP) with a mass loading of 0.6 ± 0.05 mg cm^{-2}. The electrochemical tests were performed in a three-electrode system in a membrane-free glass beaker, using Ag/AgCl electrode (with saturated KCl) and Pt foil as reference and counter electrode, respectively. Linear scan voltammetry (LSV) curves of catalysts were acquired from −0.8 V to 0.7 V vs. Ag/AgCl at a scan rate of 10 mV s^{-1}. All of the electrocatalytic reactions were conducted at ambient pressure and temperature, unless otherwise specified. All potentials measured against Ag/AgCl were converted to the reversible hydrogen electrode (RHE) scale using the following equations:

$$E_{RHE} = E_{Ag/AgCl} + E_{Ag/AgCl\ vs.\ NHE} + 0.059\ pH \qquad (1)$$

where $E_{Ag/AgCl\ vs.\ NHE}$ in eq (1) is 0.197 V at 20 °C.

The FEs of all the products were calculated based on their corresponding electron transfer per molecule oxidation using the following equations.

$$\text{Faradaic efficiency} = \frac{n_e \times n_{products} \times F}{Q} \times 100\% \qquad (2)$$

where n_e is the number of electrons required to oxidize benzyl alcohol to products. $n_{products}$ is the productivity of products, F is Faraday constant (F = 96485), Q is the quantity of electric charge.

The liquid products were quantified by high performance liquid chromatography (HPLC; Angilent 1200 Infinity Series) equipped with C18 column (Cosmosil C18-MS-II) using $MeCN/H_2O/H_3PO_4$ (40/60/0.05) as mobile phase and detected by UV detector at 220 nm.

COMSOL simulation.

The distribution of electric field intensity and surface density of reactive species on Au/CuO NPs, NSs, and NWs was modeled using COMSOL Multiphysics. The dimensions of the CuO substrates were as follows: spherical NPs with a diameter of 460 nm; hexagonal NSs measuring 550 nm in length and 17 nm in thickness; and cylindrical NWs with a height of 1000 nm and a diameter of 150 nm. Five gold nanoparticles (each with a diameter of 40 nm for NPs and NSs; and 50 nm for NWs) were uniformly embedded along the edges and faces of the CuO substrate. Spatial electric field distribution was simulated using the electrostatics module, with relative permittivity values assigned as 6.9, 18, and 80 for gold, CuO, and the solvent, respectively. A potential of 1.5 V vs. ground was applied to the gold nanoparticles, and the resulting electric field intensity was quantified based on its maximum and average values, obtained through mathematical integration over the Au/CuO volume.

The density and flux of surface reactive species (i.e., hydroxide and alkoxy) were simulated using the electrochemistry module (tertiary current distribution) with the following parameters: the bulk concentrations of the electrolyte (KOH) and reactant (ROH) were set at 1.0 M and 0.10 M, respectively. The diffusion coefficients D for potassium, hydroxide, alcohol, and alkoxy ions were 1.96×10^{-9} m^2 s^{-1}, 5.27×10^{-9} m^2 s^{-1}, 1.00×10^{-9} m^2 s^{-1}, and 1.00×10^{-9}

m^2 s^{-1}, respectively. Two single-charge-transfer electrode reactions were defined at the gold nanoparticles under a cathodic potential of 1.5 V vs. RHE, corresponding to the oxidation of hydroxide and alkoxy anions into radicals. The local current densities for both reactions were determined using Butler-Volmer kinetics, with a half-wave potential of 0.8 V vs. RHE, an exchange current density of 5.0×10^{-3} A m^{-2} and a charge transfer coefficient α of 0.5. The density of surface reactive species was estimated based on their surface concentration changes, normalized to their initial bulk concentration, within a predefined surface layer thickness of 1 nm.

Supplementary figures

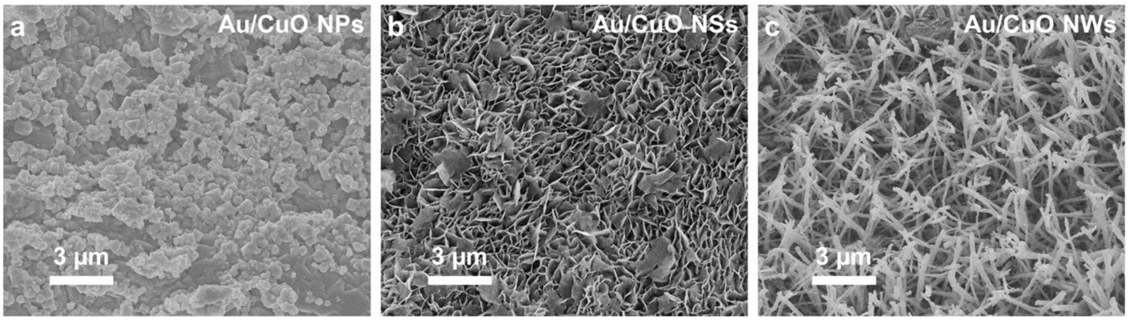

Figure S1. SEM images of (a) Au/CuO NPs, (b) Au/CuO NSs and (c) Au/CuO NWs on Cu foam.

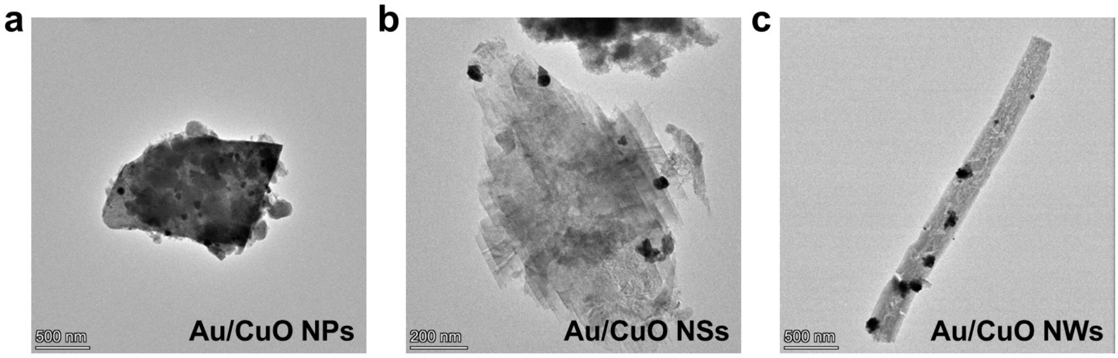

Figure S2. HRTEM images of (a) Au/CuO NPs, (b) Au/CuO NSs and (c) Au/CuO NWs.

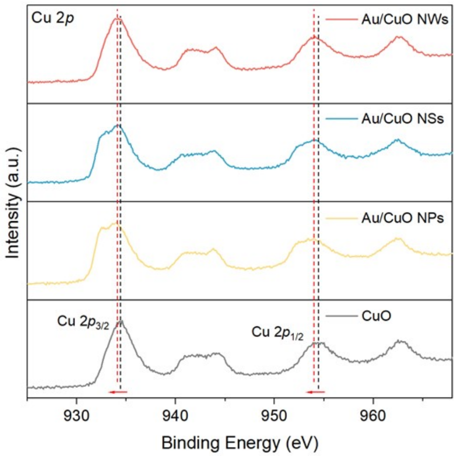

Figure S3. High-resolution Cu 2p XPS spectra of three Au/CuO cooperative catalysts and pure CuO.

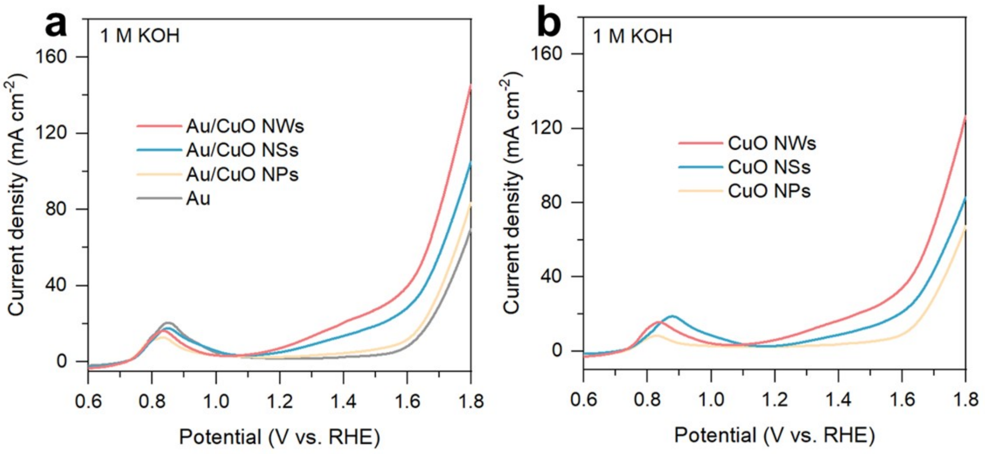

Figure S4. LSV curves of different samples on copper foam in 1 M KOH at scan rate of 10 mV s^{-1}.

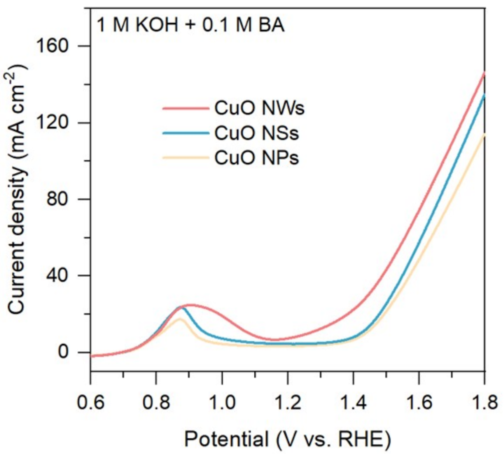

Figure S5. LSV curves of different samples on copper foam in 1 M KOH with 0.1 M benzyl alcohol over three CuO samples at scan rate of 10 mV s^{-1}.

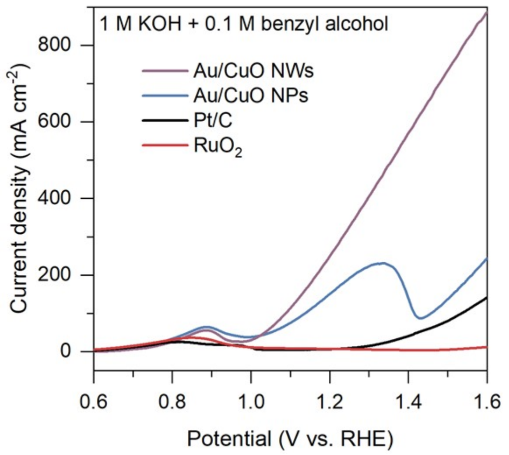

Figure S6. LSV curves of different samples in 1 M KOH with 0.1 M benzyl alcohol at scan rate of 10 mV s^{-1}.

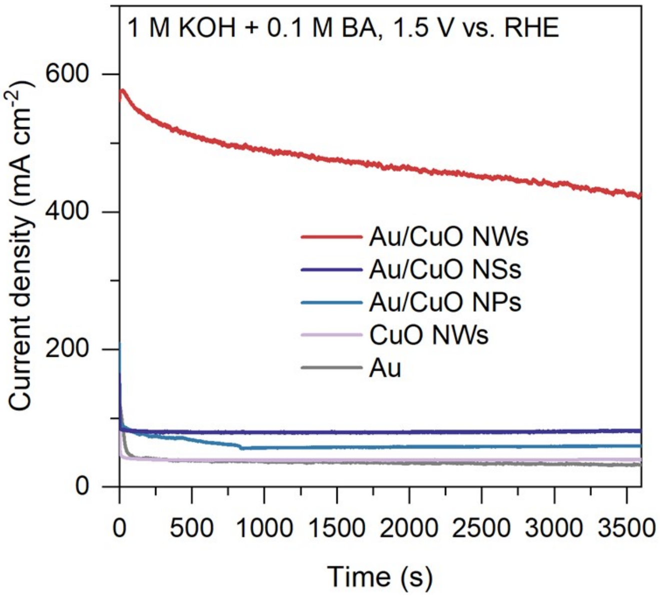

Figure S7. I-t curves of different samples at 1.5 V vs. RHE in 1 M KOH with 0.1 M benzyl alcohol (BA).

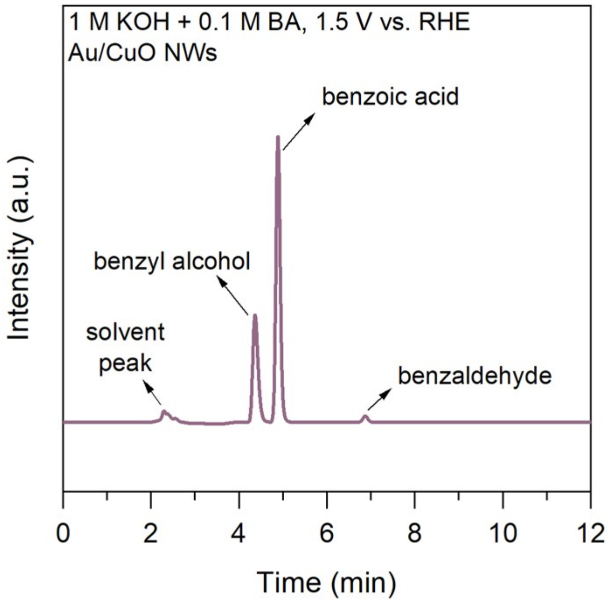

Figure S8. HPLC chromatogram of benzyl alcohol electrooxidation products over Au/CuO NWs in 1 hour.

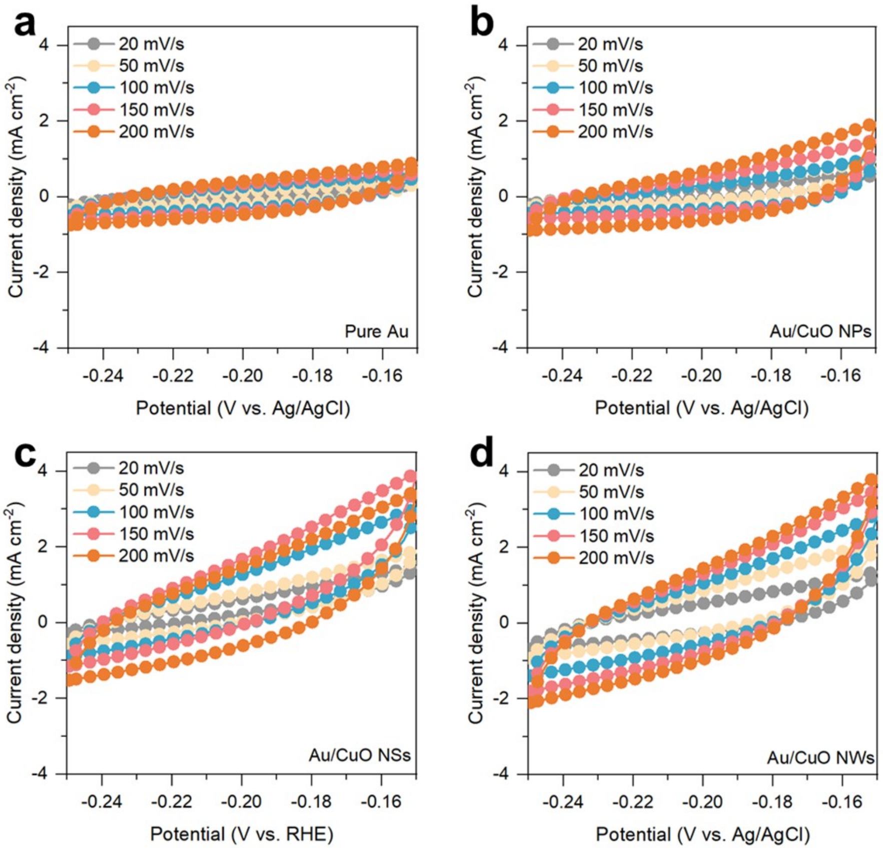

Figure S9. CV curves of (a) Au, (b) Au/CuO NPs, (b) Au/CuO NSs and (d) Au/CuO NWs measured in a non-Faradaic region of the voltammogram at the following scan rate: 20, 50, 100, 150, and 200 mV/s.[1]

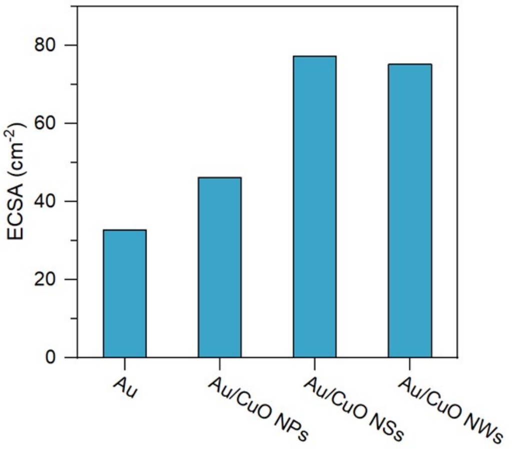

Figure S10. ECSA value of different samples.[2,3]

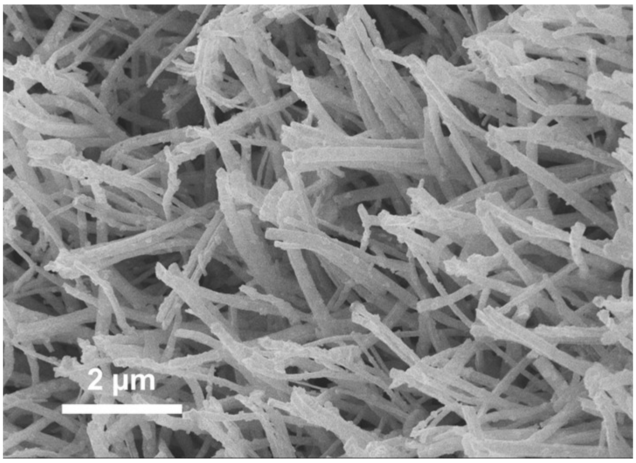

Figure S11. SEM image of Au/CuO NWs after 10 cycles reaction.

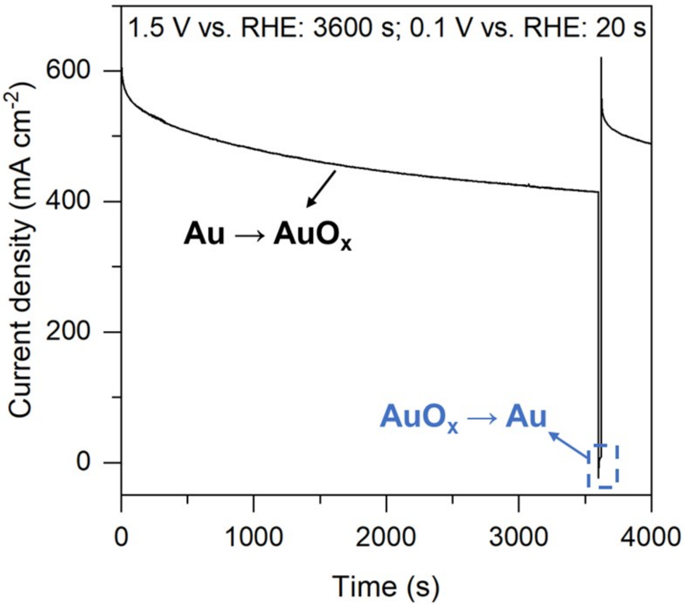

Figure 12a. I-t curves of benzyl alcohol electrooxidation over Au/CuO NWs by pulse potential strategy.

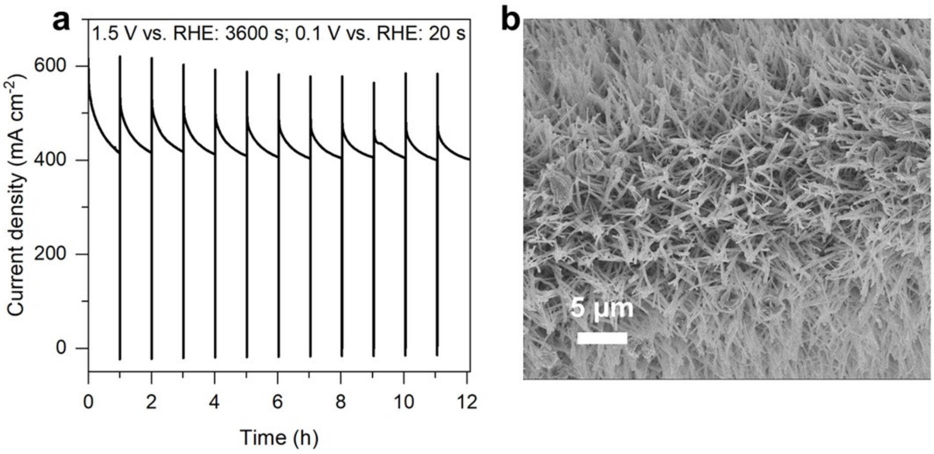

Figure S12b. (a) I-t curves of benzyl alcohol electrooxidation over Au/CuO NWs by pulse

potential strategy. (b) SEM image of Au/CuO NWs after reaction.

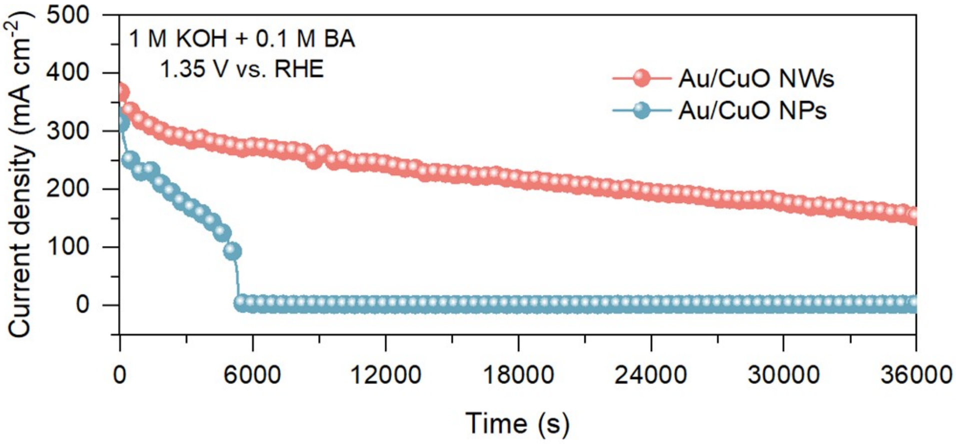

Figure S13. Current-time (*I-t*) curves of Au/CuO NPs and Au/CuO NWs in 1 M KOH with 0.1 M benzyl alcohol (BA) at 1.35 V vs. RHE.

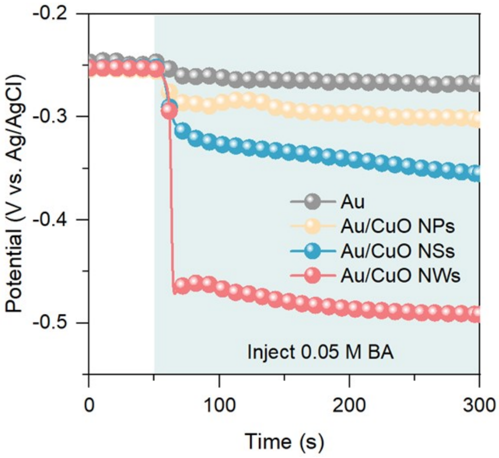

Figure S14. The OCP of different samples in 1 M KOH solution before and after benzyl alcohol (BA) injected.

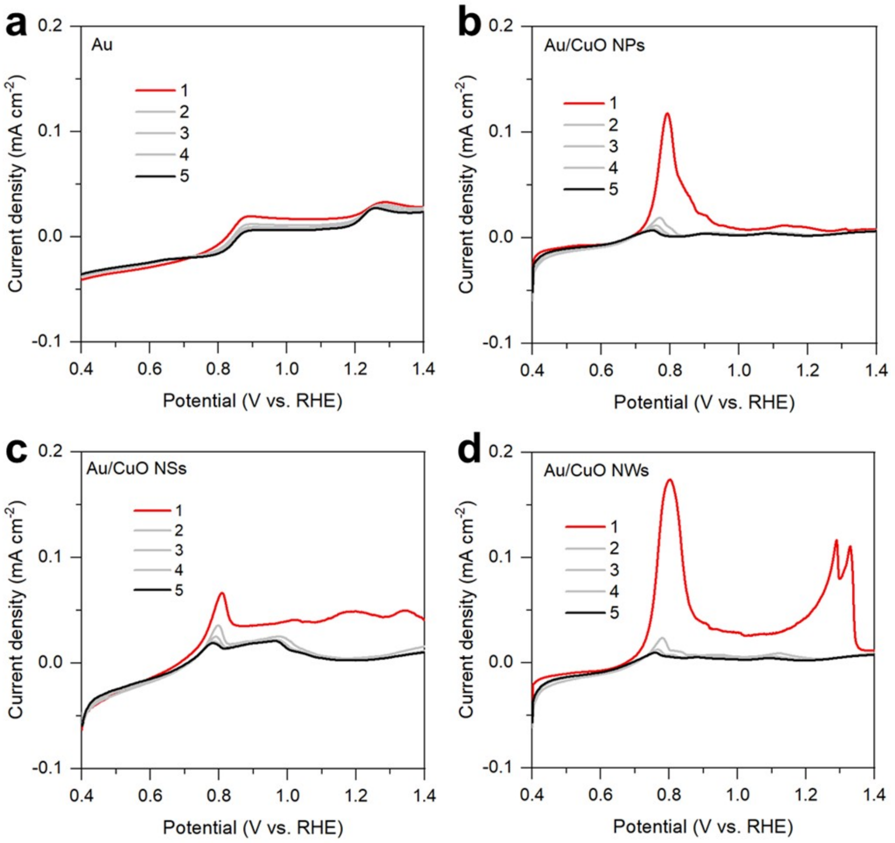

Figure S15. Adsorbate stripping from benzyl alcohol adsorption on (a) Au, (b) Au/CuO NPs, (b) Au/CuO NSs and (d) Au/CuO NWs catalysts.[4] Reaction conditions: 0.1 M KOH; scan rate = 10 mV s^{-1}. First scan (red line) and subsequent scans.

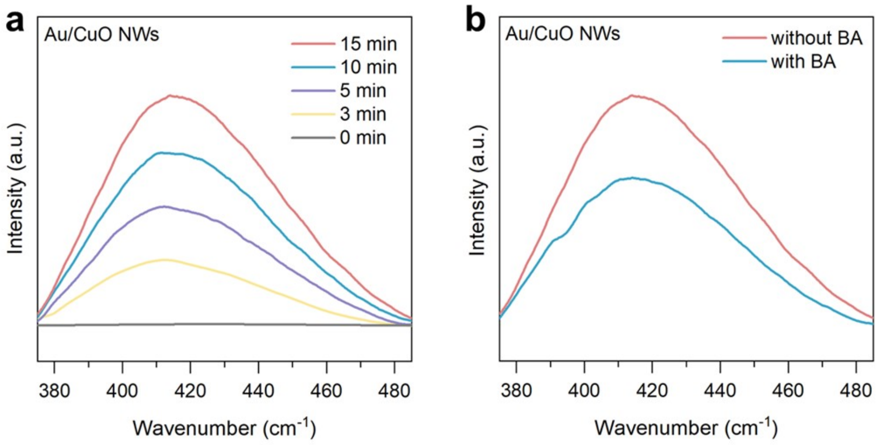

Figure S16. (a) Time-dependent fluorescence spectra of the terephthalic acid solution (4 × 10^{-4} M) in 1 M KOH with the excitation wavelength of 320 nm.[5] (b) Fluorescence spectra in 1 M KOH with and without benzyl alcohol over Au/CuO NWs.

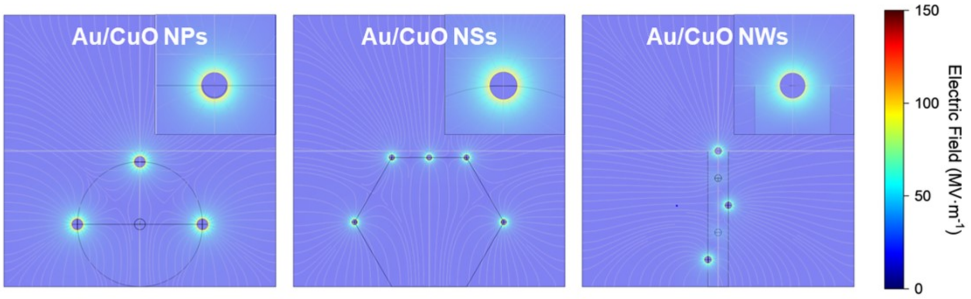

Figure S17. Electric field distribution of different Au/CuO samples through COMSOL multiple physical quantities.

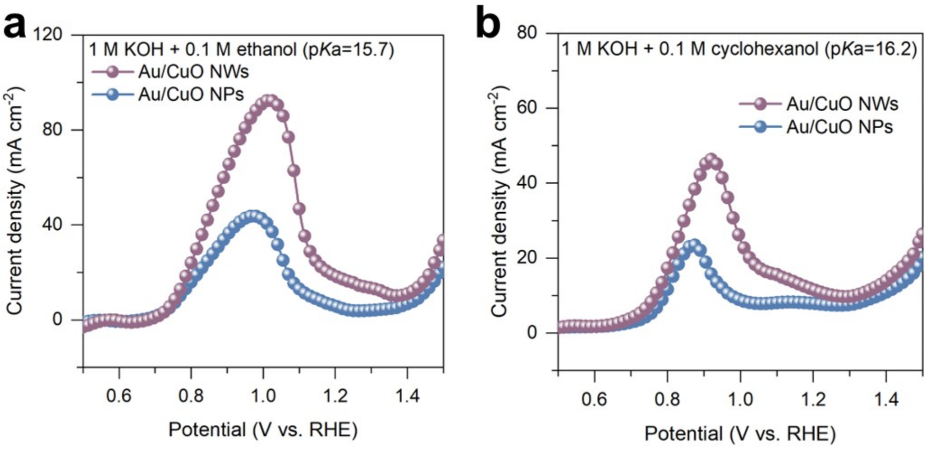

Figure S18. LSV curves of different catalysts at scan rate of 10 mV s^{-1} in 1 M KOH with (a) 0.1 M ethanol or (b) cyclohexanol.

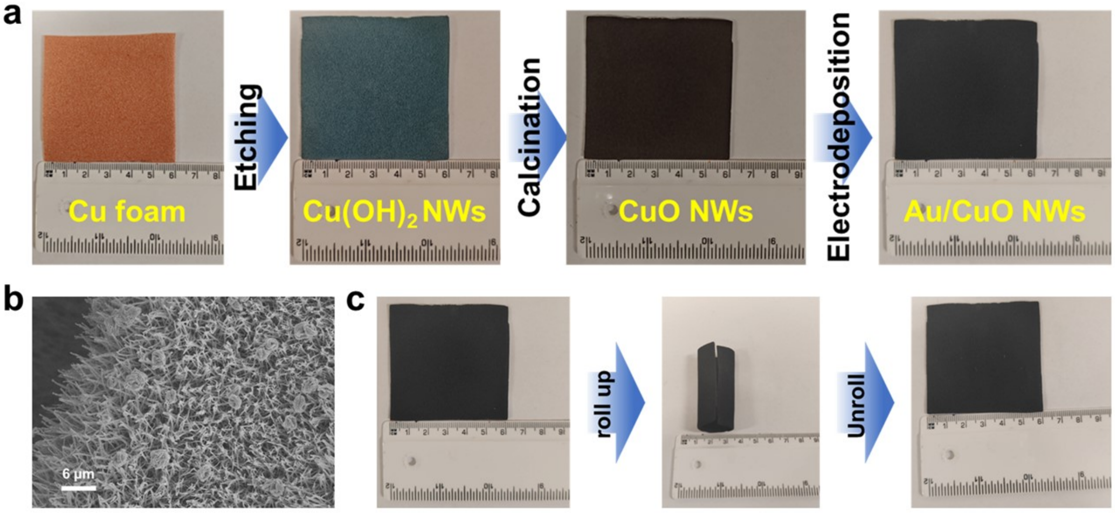

Figure S19. (a) Photographs illustrating the fabrication process of large-area Au/CuO NWs. (b) SEM image of large-area Au/CuO NWs. (c) Catalyst mechanical stability evaluation.

Supplementary Table

Supplementary Table 1. Mass loading of Au for different catalysts by inductively coupled plasma-atomic emission spectrometry (ICP-AES).

**Catalysts**	**Mass loading of Au (mg cm^{-2})**
Au/CuO NPs	0.183
Au/CuO NSs	0.179
Au/CuO NWs	0.178

Supplementary Table 2. Comparison of the catalytic performance of benzyl alcohol (BA) electrooxidation reported in the literatures and in this work.

**Catalysts**	**C_{BA}**	**Conductive substrate**	**Electrolyte**	**Scan rate of LSV (mV s^{-1})**	**Potential (V *vs*. RHE)**	**Current density (mA cm^{-2})**	**Ref.**
NC@CuCo$_2$N$_x$/CF	10 mM	carbon fiber	1 M KOH	5	1.4	125	*6*
PtZn-ZnOx	0.1 M	carbon paper	1 M KOH	50	0.8	125	*7*
A-Ni-Co-H	0.1 M	Ni foam	1 M KOH	10	1.45	400	*8*
Co_3O_4 NWs/Ti	10 mM	Ti membrance	0.1 M NaOH	10	2.6	9	*9*
Ni-OH/NF	0.1 M	Ni foam	1 M KOH	1	1.33	100	*10*
h-Ni(OH)$_2$	40 mM	-	1 M KOH	5	1.5	180	*11*
hp-Ni	10 mM	Ni foam	1 M KOH	2	1.4	100	*12*
NiCo/AC	0.1 M	Carbon paper	1 M KOH	5	1.35	60	*13*
N-Mo-Ni/NF	0.1 M	Ni foam	1 M KOH	5	1.34	100	*14*
FeCoNiAlMo/CNT	0.2 M	Carbon fiber paper	1 M KOH	5	1.4	20	*15*
NiCo(OOH)$_x$	0.1 M	Ni foam	1 M KOH	5	1.35	50	*16*
ZIF-9@xGO	0.1 M	Ni foam	1 M KOH	10	1.6	204	*17*
Au/CoOOH	0.1 M	Ni foam	1 M KOH	10	1.5	540	*18*
Au/CuO NPs					1.34	230	
Au/CuO NSs	0.1 M	Cu foam	1 M KOH	10	1.5	602	*This work*
Au/CuO NWs					1.65	933	

Supplementary References

1. C. McCrory, S. Jung, J. Peters, T. Jaramillo, *J. Am. Chem. Soc.* **2013**, *135*, 16977−16987.
2. G. Liu, M. Wang, Y. Wu, N. Li, F. Zhao, Q. Zhao, J. Li, *Appl. Catal. B* **2020**, *260*, 118199.
3. Y. Zhang, S. Chen, Y. Zhang, R. Li, B. Zhao, T. Peng, *Adv. Mater.* **2023**, *35*, 2210727.
4. A. Garcia, M. Kolb, C. Sanchez, J. Vos, Y. Birdja, Y. Kwon, G. Tremiliosi-Filho, M. Koper, *ACS Catal.* **2016**, *6*, 4491−4500.
5. F. Liu, X. Gao, R. Shi, Z. Guo, E. Tse, Y. Chen, *Angew Chem. Int. Ed.* **2023**, *62*, e202300094.
6. J. Zheng, X. Chen, X. Zhong, S. Li, T. Liu, G. Zhuang, X. Li, S. Deng, D. Mei, J. Wang, *Adv. Funct. Mater.* **2017**, *27*, 1704169.
7. R. Liu, W. Tu, A. Pei, W. Huang, Y. Jia, P. Wang, D. Liu, Q. Wu, Q. Qin, W. Zhou, L. Zhou, K. Yan, Y. Zhao, G. Chen, *J. Am. Chem. Soc.* **2025**, *147*, 10339−10348.
8. H. Huang, C. Yu, X. Han, H. Huang, Q. Wei, W. Guo, Z Wang, J. Qiu, *Energy Environ. Sci.* **2020**, *13*, 4990−4999.
9. Z. Yin, Y. Zheng, H. Wang, J. Li, Q. Zhu, Y. Wang, N. Ma, G. Hu, B. He, A. Knop-Gericke, R. Schlögl, D. Ma, *ACS nano* **2017**, *11*, 12365−12377.
10. L. Ming, X. Wu, S. Wang, W. Wu, C. Lu, *Green Chem.* **2021**, 23, 7825−7830.
11. X. Chen, X. Zhong, B. Yuan, S. Li, Y. Gu, Q. Zhang, G. Zhuang, X. Li, S. Deng, J. Wang, *Green Chem.* **2019**, 21, 578−588.
12. B. You, X. Liu, X. Liu, Y. Sun, *ACS Catal.* **2017**, 7, 4564−4570.
13. G. Liu, C. Zhao, G. Wang, Y. Zhang, H. Zhang, *J. Colloid Interface Sci.* **2018**, *532*, 37−46.
14. J. Wan, X. Mu, Y. Jin, J. Zhu, Y. Xiong, T. Li, R. Li, *Green Chem.* **2022**, *24*, 4870−4876.
15. Y. Wang, M. Ni, W. Yan, C. Zhu, D. Jiang, Y. Yuan, H. Du, *Adv. Funct. Mater.* **2024**, *34*, 2311611.
16. M. Zhang, Z. Xu, B. Liu, Y. Duan, Z. Zheng, L. Li, Q. Zhou, V. Matveeva, Z. Hu, J. Yu, K. Yan, *AIChE J.* **2023**, *69*, e18077.
17. S. Chongdar, A. Ghosh, R. Bal, A. Bhaumik, *J. Mater. Chem. A* **2024**, *12*, 233−246.
18. Z. Li, Y. Yan, S. Xu, H. Zhou, M. Xu, L. Ma, M. Shao, X. Kong, B. Wang, L. Zheng, H. Duan, *Nat. Commun.* **2022**, *13*, 147.
